# In Situ Fabrication of Metal Chalcogenide on Metal–Organic Frameworks for Enhanced Photocatalytic CO_2_ Reduction to Liquid Fuel

**DOI:** 10.1021/acsomega.6c03925

**Published:** 2026-08-19

**Authors:** Thillai Lakshmi, Shanmuga Priya Selvanathan, Lavanya Mulky, Muhammad Tahir

**Affiliations:** † Manipal Institute of Technology, 76793 Manipal Academy of Higher Education, Manipal 576104, Karnataka, India; ‡ Chemical and Petroleum Engineering Department, UAE University, Al Ain, Abu Dhabi, P.O. Box 15551, United Arab Emirates

## Abstract

A highly effective photocatalyst ought to have an adequate number of active sites, use enough photons, and transport excited electron–hole pairs rapidly. This study describes how an in situ developing approach was successfully used to produce a highly active, affordable composite. Because of its highly uniform surface, the chosen MOF permits active sites in the photocatalytic reduction activity and provides sufficient space for the chalcogenide nanoparticles to disperse. The as-synthesized chalcogenide has a larger surface area than the one that was reported previously. Additionally, in comparison to pure MOF and chalcogenide, the combination displays a higher ability to absorb light. The separation and transmission of photoexcited electron–hole pairs are accelerated by the significant reduction in the rate of photogenerated carrier recombination. Characterization techniques, including Fourier transform infrared (FTIR) spectroscopy, X-ray diffraction (XRD), ultraviolet absorption (UV), scanning electron microscopy (SEM), energy-dispersive X-ray spectroscopy (EDS), and electrochemical impedance spectroscopy (EIS), are used to thoroughly examine and analyze the morphological, compositional, structural, and optical characteristics. The distinctive peaks that corresponded to the JCPDS data stood in accordance with the XRD pattern of the synthesized semiconductor. A thorough investigation of photocatalysis under light irradiation was carried out using a simple photoreactor system. The desirable rate of ethanol production was 3730.29 μmol g^–1^ h^–1^ at a 30% addition ratio of MOF, showing improved CO_2_ reduction efficiency when compared with pure synthesized chalcogenide and MOF following a Z-scheme heterojunction.

## Graphical abstract

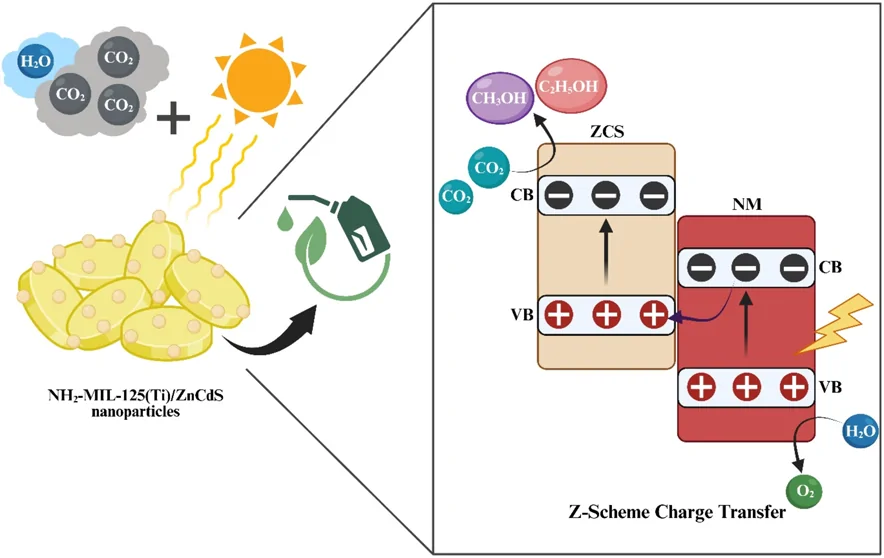

## Introduction

Carbon dioxide is one of the greenhouse gases (GHGs) that has a significant negative influence on the environment, contributing to problems such as climate change and global warming. CO_2_ emissions are constantly rising as a result of human activities such as deforestation, burning fossil fuels, transportation, industrial growth, and the production of heat and power. Conversion techniques are one of the great solutions discovered by scientists and researchers worldwide in their efforts to reduce the level of CO_2_ emissions. The photocatalytic conversion method, which uses CO_2_ to produce solar fuels, is the main focus of this research project. In order to reduce the adsorbed CO_2_ to usable solar fuels, this method uses solar energy simply from the light source and converts it into chemical energy that the photocatalyst must produce. This process is sometimes termed artificial photosynthesis since it imitates natural photosynthesis. Since its inception in the 1910s, photocatalysis has developed into a cutting-edge field.^1^ The conversion of CO_2_ into solar fuels is accelerated by using semiconductors as photocatalysts with light irradiation, where water serves as a proton source. CO can be reduced to several different products throughout the reduction process, including CO, HCOOH, HCHO, CH_3_OH, CH_4_, and C_2+_ products including C_2_H_4_, C_2_H_6_, and C_2_H_5_OH.^2^ The main elements required for photocatalysis are a photocatalyst and a photocatalytic setup that includes a photoreactor, a CO_2_ source, and a light source. The number of solar fuels produced was subsequently determined using a gas chromatograph. The goal of this research is to develop more environmentally friendly products without the use of photosensitizers, sacrificial agents, or other chemicals.

A class of materials known as metal–organic frameworks (MOFs) is made up of inorganic metal centers and organic ligands. MOF photocatalysts are superior to traditional semiconductor photocatalysts because they can be strategically constructed and improved at the molecular level using various combinations of metal-oxo clusters, bridging, and linking organic linkers. Furthermore, reactants and products can more easily permeate through the open framework structures of MOFs due to their intrinsic porosity.^3^ MOF-derived photocatalysts still have some problems, including their low quantum efficiency and low stability. To increase the photocatalytic activity of MOFs, a number of methods are employed, such as metal ligand replacement and noble metal deposition. Semiconductor-MOF heterostructures such as NH_2_-MIL-101­(Fe)@Bi_2_MoO_6_,^4^ NH_2_-MIL-101­(Fe)/CdS/g-C3N_4_,^5^ rGO/NH_2_-MIL-125­(Ti),^6^ and MoS_2_/UiO-66@CdS^3^ have demonstrated good activity recently. Band gap engineering can be achieved by varying the concentration of amino functionalities in the linker.^7^ By carefully adjusting the energy levels of both HUMO and LUMO, the required band gap can be obtained.^8^

Chalcogenides are currently among the most efficient photocatalysts utilized in photocatalysis applications because of their very small band gap and reduced recombination, which allow them to effectively utilize visible light.^9^ Because of their improved light absorption capabilities, efficient charge separation, and tunable band gap, ZnCdS-based photocatalysts are growing successful in converting CO_2_ into solar fuels. With production rates of 169.19 and 188.23 μmol g^–1^ h^–1^, respectively, ZnS/CQDs/NH_2_-MIL-125­(Ti) demonstrated good liquid products, including methanol and ethanol.^10^ Cu_2_O/Pt/NH_2_-MIL-125­(Ti) generated two liquid products such as CH_3_OH (192 μmol g^–1^ h^–1^) and CH_3_CH_2_OH (307 μmol g^–1^ h^–1^).

The photocatalytic reduction of CO_2_ to ethanol and methanol is studied in this work utilizing photocatalyst composites composed of amino-functionalized MOF (NH_2_-MIL-125) and solid solution metal sulfide (Zn_0.2_Cd_0.8_S). The surface area of the as-synthesized Zn_0.2_Cd_0.8_S is more than previously reported. A comparable composite has also been reported for several ratios of Zn_*x*_Cd_1–*x*_S, with the best hydrogen generation rate from Zn_0.5_Cd_0.5_S/MIL-125-NH_2_(Ti) being 92,498 μmol g^–1^ h^–1^.^11^ Therefore, the generation of liquid fuel was the main focus of this research initiative. Initially, the parameters for the synthesis of pristine materials were modified and examined appropriately. After that, the synthesis technique carried out was the in situ method to synthesize the composites. After the MOF % was suitably adjusted, the photocatalytic activity and stability were studied. We anticipate that the current work will raise interest in the development of innovative high-performance chalcogenide-MOF composites for liquid production by employing chalcogenides and MOFs.

## Experimental Section

### Materials

Cd (CH_3_COO)_2_·2H_2_O (98%, extra pure) and titanium isopropoxide are obtained from Spectrum Reagents and Chemicals Pvt. Ltd., and 2-aminoterephthalic acid (>98%) is purchased from TCI Chemicals. Zn (CH_3_COO)_2_·2H_2_O (99.99%), thiourea (98%, extra pure), ethylene glycol, poly­(vinyl pyrrolidone) K25, DMF, and CH_3_OH were purchased from Loba Chemie Pvt. Ltd. Methanol and acetone were purchased from Actylis Finar Limited.

### Synthesis of Zn_0.2_Cd_0.8_S/NH_2_-MIL-125­(Ti)

The synthesis of pristine NM and ZCS is discussed in the Supporting File. As seen in Figure 1, the NZ composites were developed using an in situ technique. The synthesis process was adjusted in accordance with the findings of Su et al., who used an in situ approach to synthesize Cd_0.2_Zn_0.8_S/UiO-66-NH_2_. This was then evaluated for photocatalytic reduction of CO_2_, which ultimately yielded methanol.^12^ In the in situ approach, 40 mL of ethylene glycol containing poly­(vinyl pyrrolidone) was used to dissolve cadmium acetate, zinc acetate, and thiourea. The mixture was then agitated at room temperature. In the meantime, a known quantity of NM was extracted by sonicating 20 mL of ethylene glycol for half an hour. The combined salt solution was gradually mixed with the NM solution and stirred. We added thiourea. The resultant solution was autoclaved at 150 °C after being agitated and sonicated for 15–20 min. After centrifuging the product to separate and gather it, it was repeatedly washed with acetone. At 60 °C, the final product was dried. The final products are labeled as 10NZ to 50NZ.

**Figure 1:**
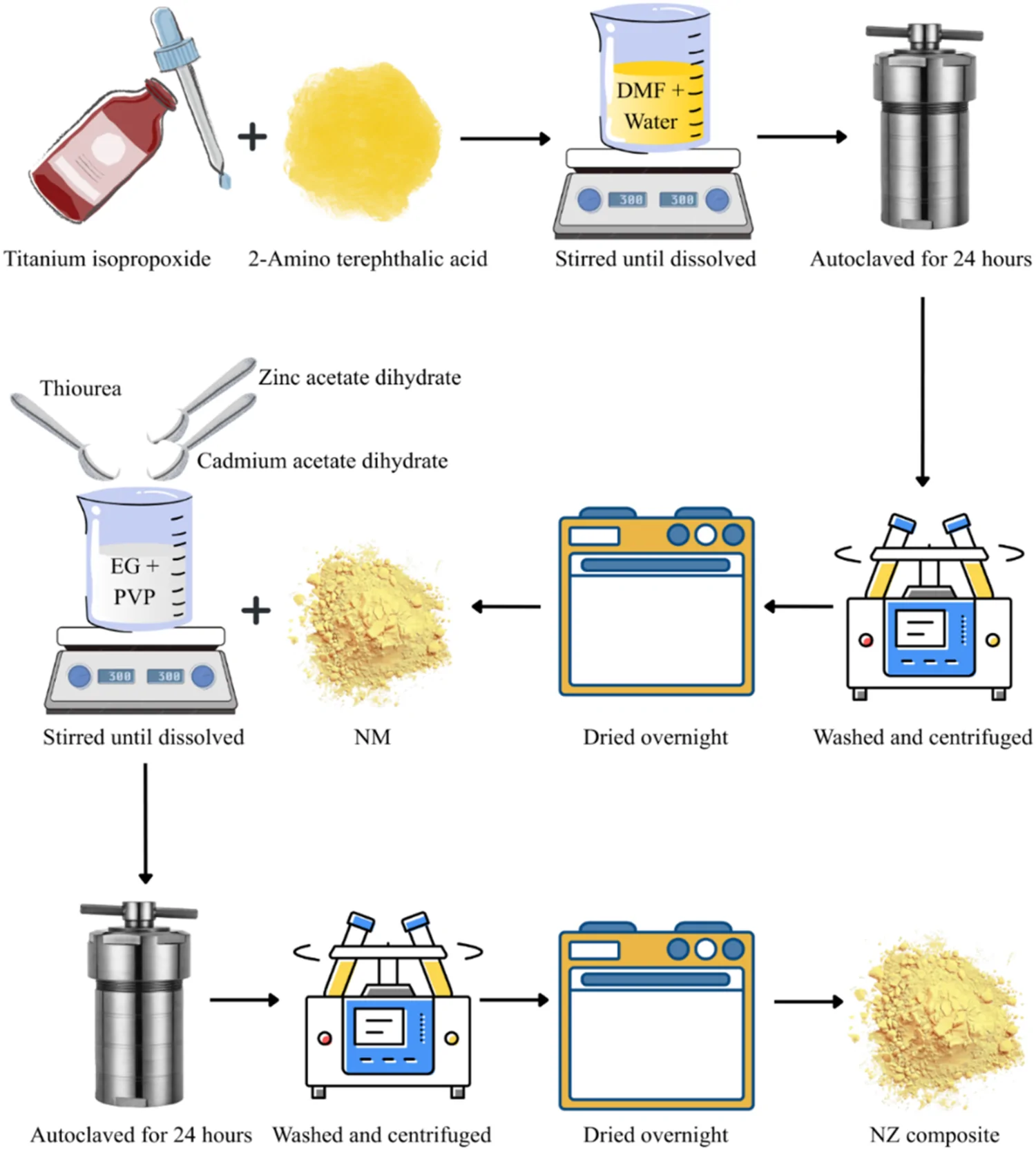
Illustration of preparation of Zn_0.2_Cd_0.8_S/NH_2_-MIL-125­(Ti) composites (image drawn by Thillai Lakshmi).

### Characterization

Cu K radiation from an X-ray diffractometer (Empyrean third Gen, Malvern PANalytical) was used to analyze the crystal structure of the pristine and composites. The microstructures and morphologies of the samples were examined using a scanning electron microscope equipped with an energy-dispersive X-ray (EDX) spectrometer. The X-ray photoelectron spectroscopy (XPS) measurement was carried out using the Thermo Fisher Nexsa XPS apparatus with Al K α source, a vacuum of 10^–8^ mbar, a pass energy of 200 eV, a dwell time of 10 ms, and a binding energy scale ranging from 0 to 1350 with a hemispherical analyzer, and an XPS work function value of 4.2 eV. For static adsorption methods, the Smart Sorb 92/93 model was employed, and surface areas and pore size distributions were computed using the Brunauer–Emmett–Teller (BET) method. A Lambda 950 PerkinElmer UV–vis spectrophotometer with a wavelength range of 175–3300 nm was used to record the materials’ UV–vis absorption spectra. Photoluminescence (PL) was measured using Varian Cary Eclipse Photo Luminescence Spectrophotometer with an Oxford low temperature using LN_2_ 77K set up to high temperature 100–373 K configuration. Electrochemical impedance spectroscopy (EIS) studies were conducted using a potentiostat (CH600 d-series, US model with CH-instrument β software).

### Photocatalytic Activity

The photocatalytic activity was measured in a lab-scale setup by using a CO_2_ cylinder as the CO_2_ source. The gas flow was controlled with a mass flow controller. A UV lamp was employed as a light source in a closed, airtight chamber (conical flask), a gas circulation system, and a cooling jacket to reduce the lamp’s heat output. H_2_O and CO_2_ were combined by bubbling CO_2_ in a water bubbler and feeding it straight to the reactor chamber. In each run, 0.1 g of photocatalyst was dispersed in an adequate volume of aqueous solution containing water and solvents, such as 0.1 M NaOH. To prevent outside light from interfering with the reactor, a tinfoil was placed over the unit. Before irradiation, the solution was saturated with CO_2_ and bubbled through the reactor for half an hour to eliminate the dissolved oxygen. First, a blank test was conducted without CO_2_, and then without a light source. This is to demonstrate that the products occur only when CO_2_ and a light source are present. The suspension was then exposed to light that is apparent and magnetically agitated. Using a syringe, 1 mL of the resultant product was collected at set intervals and centrifuged to eliminate the photocatalyst. Liquid product concentrations were measured using an AUTOGC gas chromatography system, model 1100, equipped with a polar column and an FID detector.

## Results and Discussion

### Zn_0.2_Cd_0.8_S Solutions

XRD analysis gives information on the crystal structure of the photocatalysts synthesized. To prove that the chalcogenides synthesized are solid solutions rather than a physical mixture of ZnS and CdS, this analysis is performed for crystallographic confirmation. Figure 2(a) depicts the XRD patterns of the ZCS series, such as Zn_0.2_Cd_0.8_S, Zn_0.5_Cd_0.5_S and Zn_0.8_Cd_0.2_S, where the Zn and Cd metal ratios are varied accordingly during the synthesis procedure. The diffraction peaks of pure Zn_0.8_Cd_0.2_S are at 27.75°, 45.262°, 49.873°, and 53.67°, hence there are different 2θ values compared with the peaks of CdS (JCPDS 49–1302) and ZnS (JCPDS 05–0566), as shown in Figure 2(a).^13^ It can be observed that as the Zn mole fraction increases in the ZCS chalcogenide, there is a shift of peak (111) at 27.75° toward the right direction. The peaks (200), (220), and (311) at 45.262°, 49.873°, and 53.67° also show a shift from left to right direction. This demonstrates the substitutional doping of the Zn metal ion into the CdS lattice. Since the Zn metal ion has a smaller atomic radius than the Cd metal ion, when Zn is doped, there is a contraction in the lattice, resulting in smaller atomic parameters and a smaller interplanar spacing (*d*).^14^ Since 2θ is inversely proportional to the interplanar spacing (*d*) according to Bragg’s law, a smaller *d* corresponds to a larger 2θ, so we can observe the gradual shift in the peaks from left to right as the Zn metal ratio increases. This implies that ZCS is not physically mixed or compounded with ZnS and CdS, rather forms solid solutions.^13^ The peak (220) at 49.873° intensity has decreased along with a shift toward the right, and it is not present in the Zn_0.8_Cd_0.2_S XRD pattern. The decrease and disappearance of the peak indicate structural deterioration, which has occurred instead of simple contraction in the lattice due to the increase in the Zn metal ion ratio; the atomic substitution has disrupted the distinct planes. This pattern is in line with Vegard’s law, which suggests the formation of a homogeneous structure.^14^ The ZCS series were tested for photocatalytic reduction of CO_2_ for liquid fuel production, and it was found that, except for Zn_0.2_Cd_0.8_S, there was no products formed, hence Zn_0.2_Cd_0.8_S was used for further research.

**Figure 2:**
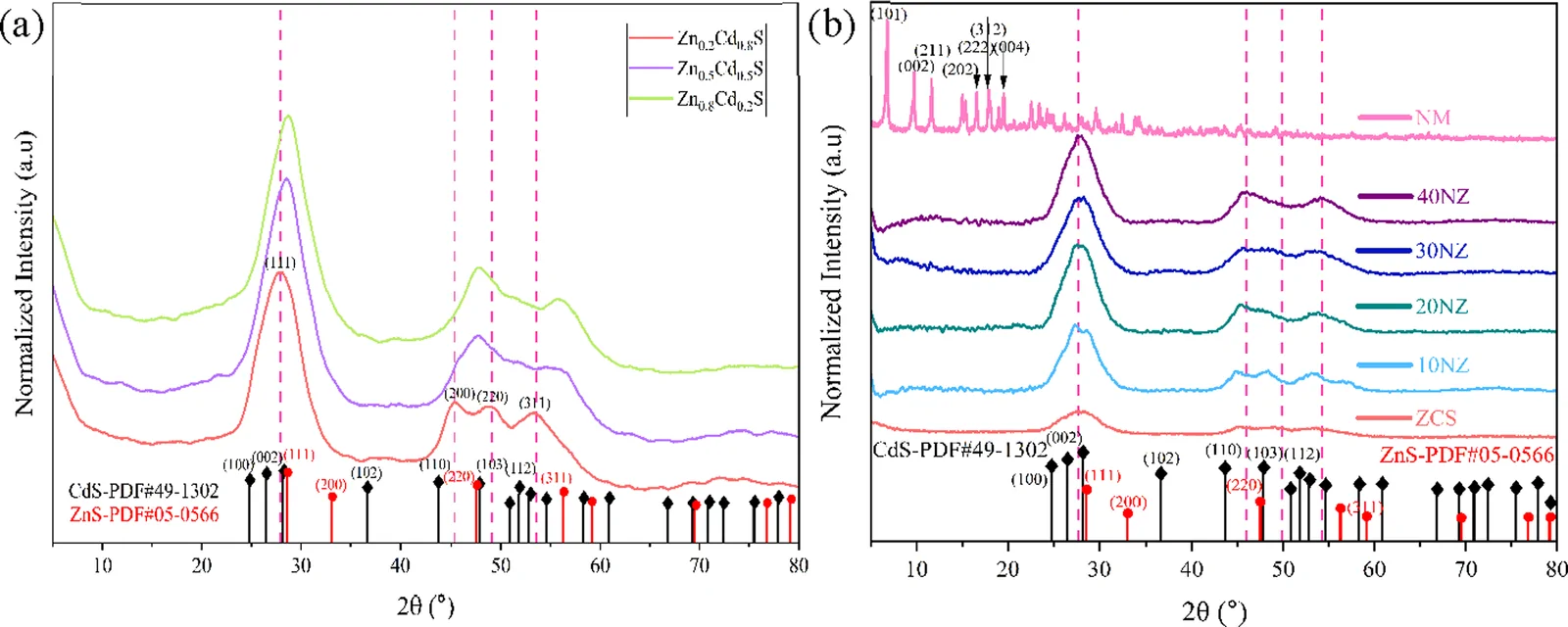
XRD patterns of (a) ZCS series and (b) NM, ZCS, and NZ with different weight contents of NM.

### Zn_0.2_Cd_0.8_S/NH_2_-MIL-125­(Ti) Composites

In Figure 2(b), the observed XRD for NM and the XRD pattern from the literature^15^ have the same peak range, two theta values, peak type, and peak count. According to the literature, the NM exhibited specific signal peaks at 6.78°, 9.73°, 11.65°, 14.98°, 16.58°, 17.91°, 18.97°, and 19.55°, similar to those shown in Figure 2(b). The XRD patterns of NM matched well with the JCPDS file no. 96–721–1160. The crystalline size of the NM was calculated to be 37.7 nm. At a 2θ value of 6.7865°, the FHWM was 0.198, and the lattice strain was calculated to be 0.006, which means there is no distortion present in the crystallite lattice. When the amount of NM was less than 40 wt %, the XRD patterns of NZ composites did not display any peaks of NM, which may be due to the low content of NM. The composite peaks have a clear increase in their intensity as the percentage of NM increases, which may be due to the increased concentration of NM; this also indicates that the amorphous nature of ZCS is showing crystallinity due to the presence of crystalline NM, and as the content of NM increases, its crystallinity increases. If the content of NM is increased, there might be a peak visibility of NM in the composites’ XRD.

The morphology and microstructures, along with the composition, of the pristine and the composites were analyzed using SEM and EDX analyses. Cheng et al., in their work, showed that the variation in the volume ratio of CH_3_OH to DMF is effective in modifying the (001) to (111) facet ratio,^16^ and the ratio was maintained at 1­(CH_3_OH):9­(DMF) throughout the synthesis procedure in this research work. Similarly, Hu et al. proved that the reactant concentration modification during crystallization can vary the morphology from a circular plate to a tetragon and to an octahedron and by SEM analysis they proved that the changes in their morphology occurred, whereas the XRD patterns for all of the samples remained the same without showing any changes in their crystal structure.^17^ Figure 3(a,b) shows that the MOF formed has a circular disk shape, circular rock-like structure, exposing the (001) facet on the top and bottom; hence, the NM synthesized is of (001) facet. The SEM images in Figure 3(c,d) show that the ZCS particles are spherically shaped, which are identical to each other and are of similar size and the same diameter, ranging from 215 to 220 nm. Figure 3(e,f) shows that the ZCS is well dispersed on the surface of NM, where we can observe that the original morphology of NM has some modifications because the ZCS spherical structure binds well with it, hiding the original circular disc structure of NM. The in situ method is used for NZ composite preparation, where the SEM images in Figure 3(e,f) prove the composite formation and the ZCS grown all over the surface of NM. To further confirm the formation of 30NZ composites, EDX elemental mapping was obtained. All of the elements that are present, such as C, N, Ti, O, Zn, Cd, and S, are well distributed in 30NZ, as seen in Figure 3(g,h). These results clearly show that NZ nanocomposites with improved heterojunction interface interaction were successfully synthesized.

**Figure 3:**
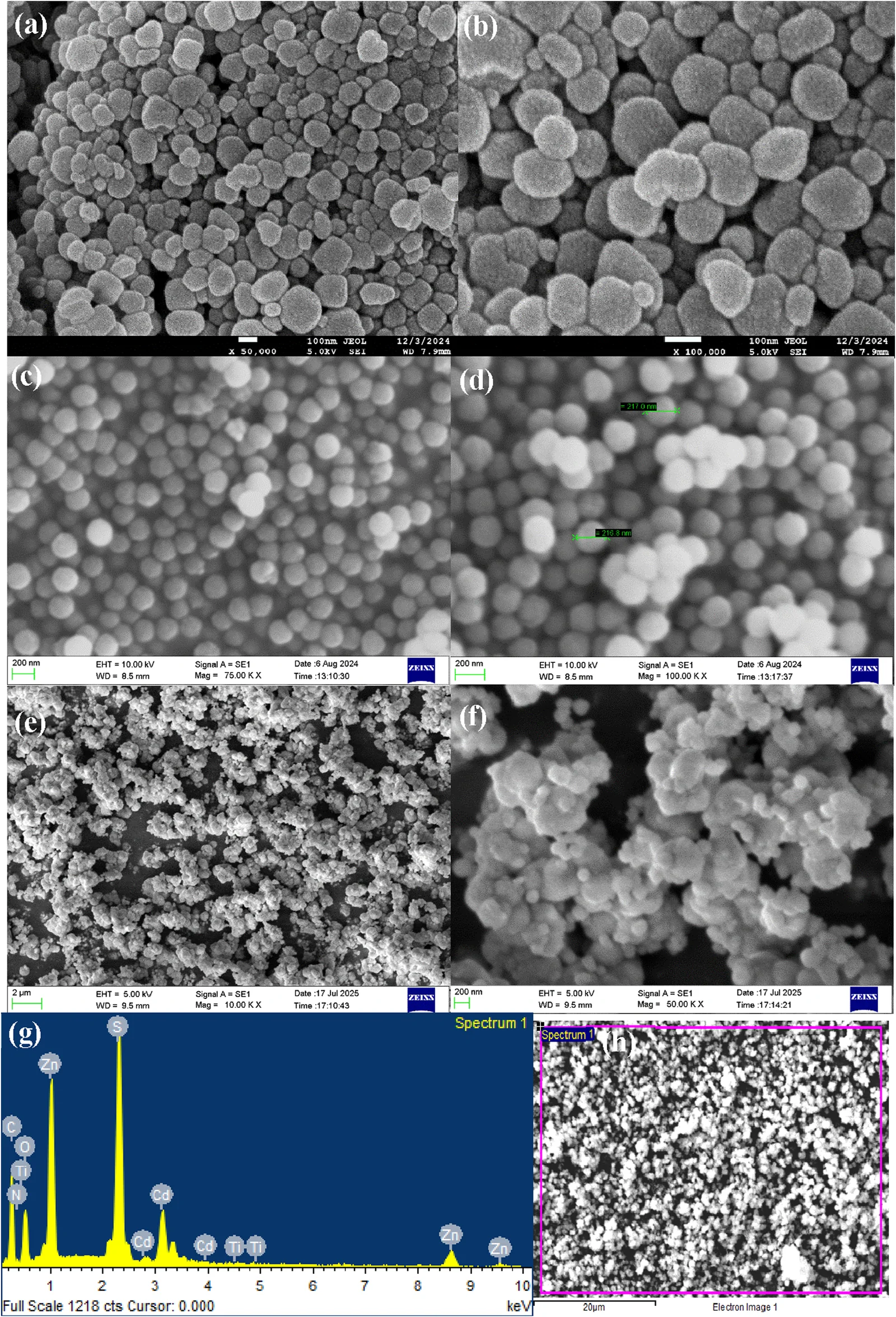
SEM images of (a, b) NM, (c, d) ZCS, (e, f) 30NZ, and (g, h) EDX mapping images of 30NZ.

A unique aspect of MOFs is their large surface area and porosity. Their microporous structures offer a wide range of pore diameters and topologies, as well as high surface areas and huge pore volumes, which have prompted extensive research into their potential for gas separation, conversion, and storage.^17^ To know the porosity and surface area of the as-synthesized pristine catalysts, BET analysis was performed and studied. The *S*_BET_ values for the pristine samples are 1065.01 and 236.91 m^2^ g^–1^ for NM and ZCS, respectively. The pore volume values are 0.6204 and 0.4627 ccg m^–1^ for NM and ZCS, respectively. Jiepeng Zhao et al. synthesized NM for CO_2_ photoreduction and obtained a surface area of 1378 m^2^ g^–1^,^18^ and Jun Zhou et al. reported the surface area of NM to be 908.60 m^2^ g^–1^.^10^ From the literature, we can say that the surface area of NM ranges from 900 to 1500 m^2^ g^–1^, as reported for CO_2_ photoreduction applications. Similarly, for ZCS, the surface area reported ranges from 20 to 100 m^2^ g^–1^,^12,13^ and the surface area reported in this research work has an improved surface area of 236.91 m^2^ g^–1^, which is one of the reasons for enhanced photocatalytic activity and efficient product yield.

UV–vis spectroscopy was used to analyze and investigate the pristine and composite materials’ capacity to absorb light in the 200–800 nm range. The literature^14^ states that pure CdS has a significant absorption edge that rises at around 520 nm, whereas pure ZnS has an absorption edge at about 350 nm. As seen in Figure 4(a), the absorption edges of the ZCS solid solution are situated between those of CdS and ZnS. It demonstrates that the sulfides produced are solid solutions.^14^ It is evident that the NM has the broader absorption band edge at 505 nm, while the ZCS absorption edge is situated at 460 nm. NZ composites’ absorption range is in between that of the pure catalysts. ZCS dispersion in the composite is shown to result in a decrease in absorption intensity. The following formula was used to compute the band gap: Using (α*h*v)^2^, where hv and α stand for photon energy and absorption coefficient, the intrinsic band gaps of bare ZCS and NM were 2.75 and 2.52 eV, respectively, as illustrated in Figure 4(b). The composites’ band gaps fall approximately in between those of the pure compounds. Electrons are more easily excited to the conduction band under illumination because of their small band gaps.

**Figure 4:**
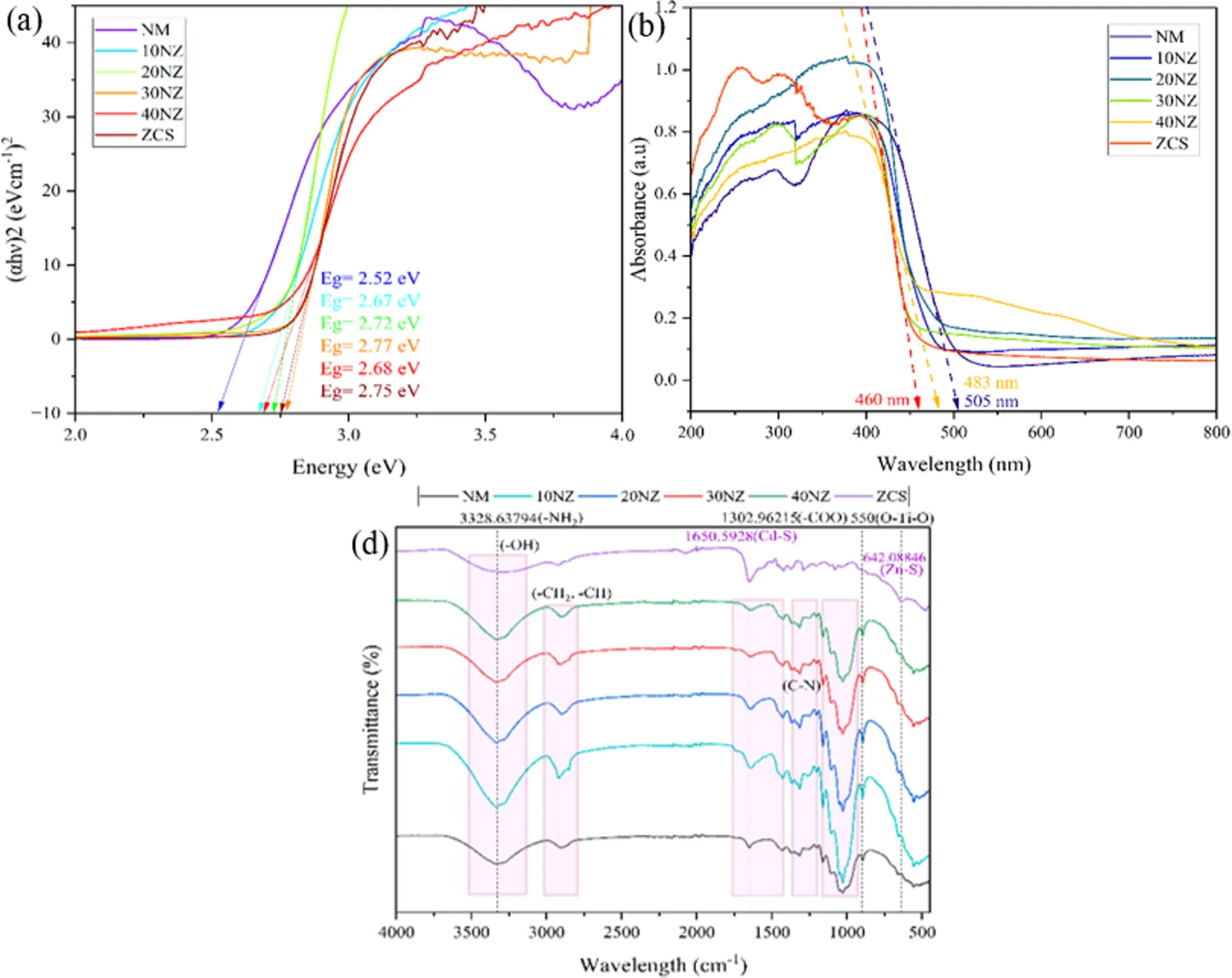
(a) UV–vis absorption spectra, (b) Tauc plots, and (c) Fourier transform infrared (FTIR) spectroscopy of NM, ZCS, and NZ composites.

Fourier transform infrared (FTIR) analysis, as shown in Figure 4(c), was conducted to elucidate the functional groups and chemical bonds of the synthesized photocatalysts. The absorption peaks at 3490 to 3200 cm^–1^ are O–H vibrations, and from the literature, it is found that the symmetric and asymmetric stretching vibrations of the primary amine are at 3413 cm^–1^, which have overlapped with the O–H vibrations. The peaks at 1610 cm^–1^ correspond to the bending vibration of the N–H bond, and 1250 cm^–1^ is associated with the stretching vibration of the N–C bond present in the aromatic ring. The peaks at 1565 and 1474 cm^–1^ represent the asymmetric stretching vibrations of −COO. The peaks in the range of 1450–1720 cm^–1^ are commonly associated with C = O present in carboxylate functional groups.^19^ The peak at 896 cm^–1^ is assigned to the bending of C–H in the organic linker. The absorption peak at 550 cm^–1^ is associated with the vibrational frequency of the O–Ti–O bond.^10,19^ The typical absorption of Cd–S stretching present in ZCS is at 1650 cm^–1^, and 642 cm^–1^ corresponds to Zn–S stretching.^20^

To study the chemical composition, surface chemical states, and the electron configuration of the NZ composite, X-ray photoelectron spectroscopy (XPS) is performed. Using the photoelectric effect, the surface of the photocatalyst is studied, where the highest valence state can be found. According to the survey spectrum in Figure 5(a), the presence of elements such as C, N, O, Ti, Zn, Cd, and S is confirmed. The peak at 284.83 eV was identified as C 1s, corresponding to the C–C, C=C, and C–H bonds of aromatic rings present in the NM of 30NZ composite. The peak, as shown in Figure 5(b), also indicates the presence of adventitious carbon, which is used as the reference material for calibration; hence, the intensity of the peak is high. Two peaks of N 1s from Figure 5(c) at 399.2 and 401.7 eV represent the –NH_2_ and –N^+^ or –NH–^+^, respectively.^10^ The presence of N from –NH_2_ and C–N or C–C from the aminoterephthalic organic linker acts as Lewis base sites that will act electronegatively while interacting with the electrophilic carbon of the CO_2_ molecule, hence enhancing the photocatalytic activity by stronger electron transfer. The peaks at 532.25 and 531.3 eV correspond to surface-adsorbed oxygen (O_β_) and lattice oxygen (O_α_), which facilitate CO_2_ adsorption through Ti–O–C bond formation (−C from CO_2_), as shown in Figure 5(d).^16^ From the literature, the exhibited peak at 532.25 eV represents the −OH group^19^ and at 531.3 eV represents the aliphatic N–C=O, respectively.^21^ The two signal peaks at 464.9 and 459.08 eV in Figure 5(e) correspond to Ti 2p_1/2_ and Ti 2p_3/2_, respectively, where Ti is present in the +4 valence state within the Ti–O cluster, which validates the presence of Ti–O clusters on the surface. Ti^4+^ present in NM acts as active metal sites and hence promotes strong interfacial interactions by the formation of a heterojunction with the semiconductor ZCS. The Zn 2p XPS spectrum (Figure 5(f)) has two peaks at 1044.9 and 1021.8 eV, which correspond to Zn 2p_1/2_ and Zn 2p_3/2_ with a valence state of +2.^22,23^ The peak at 411.9 eV corresponds to Cd 2p_1/2_ and the remaining 2 peaks at 405.5 and 404.9 eV represent Cd 2p_3/2_ in two different valence states, Cd^2+^ and Cd^0^, as shown in Figure 5(g).^24^ The two signal peaks at 163.03 and 161.8 eV in Figure 5(h) correspond to S 2p, respectively.^11^ The metallic Cd is formed due to the partial reduction of Cd^2+^ during synthesis, at certain elevated temperatures and in an electron-rich environment. The Cd^0^ acts as an electron sink, hence facilitating charge separation and contributing to enhanced photocatalytic activity.^25^ The combination of elements having d^0^ cationic configuration (such as Ti^4+^) and d^10^ cationic configuration (such as Cd^2+^ and Zn^2+^) leads to heterogeneous chemical environments that substantially enhance photocatalytic activity.^26^ This aligns well with the experimental results where the composite NZ (possessing d^0^ + d^10^ cationic configuration) has shown better yield than the pristine material. In recent studies, Hidalgo-Jiḿenez et al. worked on active catalysts incorporating d^0^ + d^10^ + s^0^ cationic configuration. Their work consists of the combination of extremely different electronegative elements alongside d^0^ + d^10^, which showed better charge transfer and enhanced yield production than the catalysts with d^0^ configuration.^27^ The d^10^ elements (such as Cd^2+^ and Zn^2+^) enhance the charge transfer, whereas the d^0^ elements (such as Ti^4+^) act as active sites for reactant absorption.^28^ Hence, mixed cationic configuration enhances the chemical reactions by facilitating efficient charge separation and transfer, making it highly beneficial for catalytic applications.^27^

**Figure 5:**
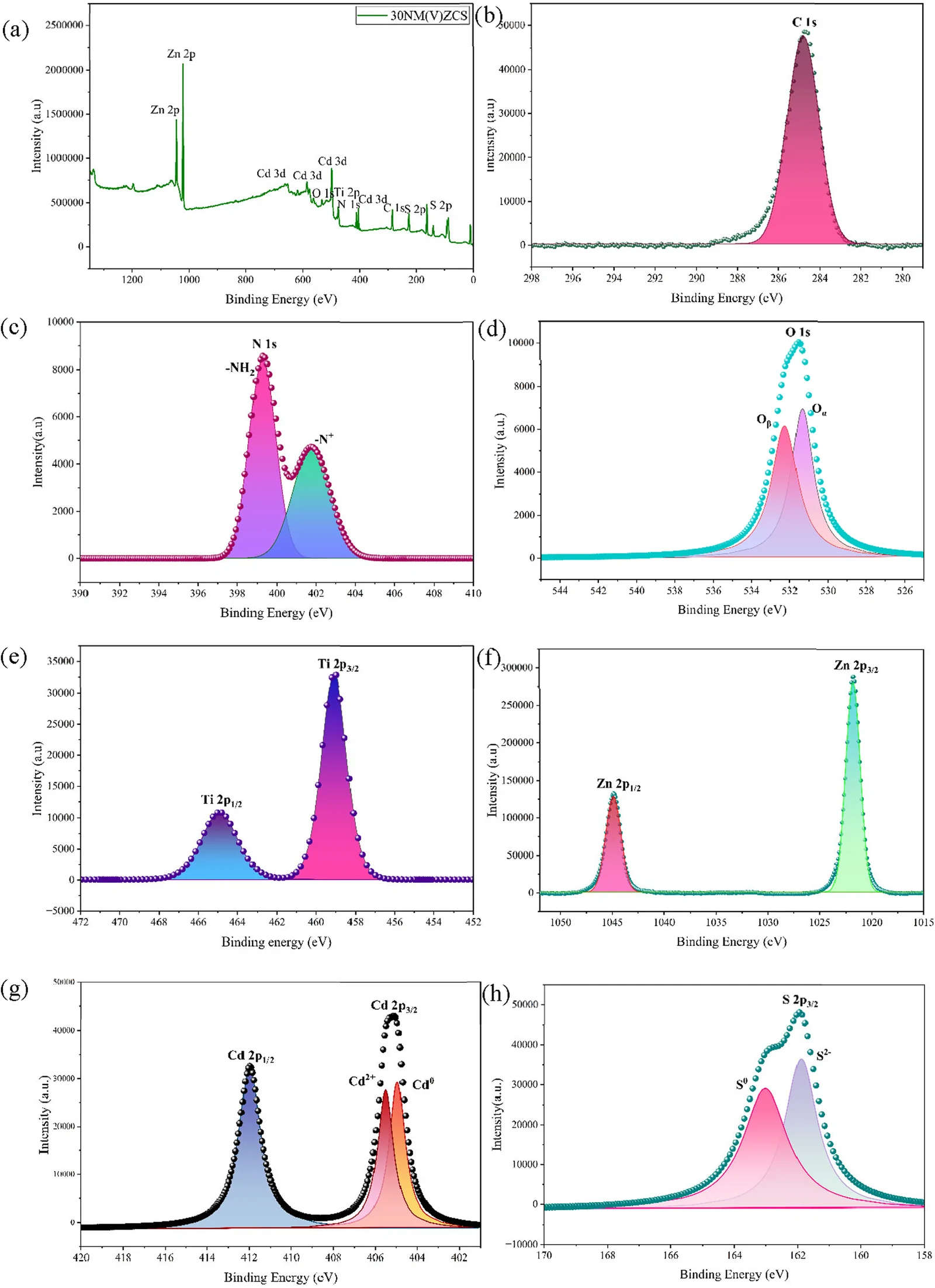
X-ray photoelectron spectroscopy of 30NZ: (a) survey, (b) C 1s, (c) N 1s, (d) O 1s, (e) Ti 2p, (f) Zn 2p, (g) Cd 3d, and (h) S 2p.

These results confirm that the composite is successfully synthesized without any changes in the chemical composition, which would have directly affected the band gap formation between the composite and its redox ability, which is very much essential for photocatalytic activity.

PL analysis is used to verify the photocatalytic activity by examining the rate at which electrons and holes within the photocatalysts recombine. As seen in Figure 6(a), PL analysis was performed on both pure ZCS and the most effective photocatalyst. Increased activity and better charge separation are typically linked to lower PL intensity. The ZCS has a noticeably higher intensity than the 30NZ composite between 450 and 700 nm, which is consistent with the experimental data. This high intensity suggests that photogenerated electrons and holes recombine rapidly; however, the addition of NM in 30NZ reduced electron–hole recombination, which is beneficial for CO_2_ photoreduction. Consequently, the composite’s lower peak intensity suggests better charge separation than pristine ZCS.^23^

**Figure 6:**
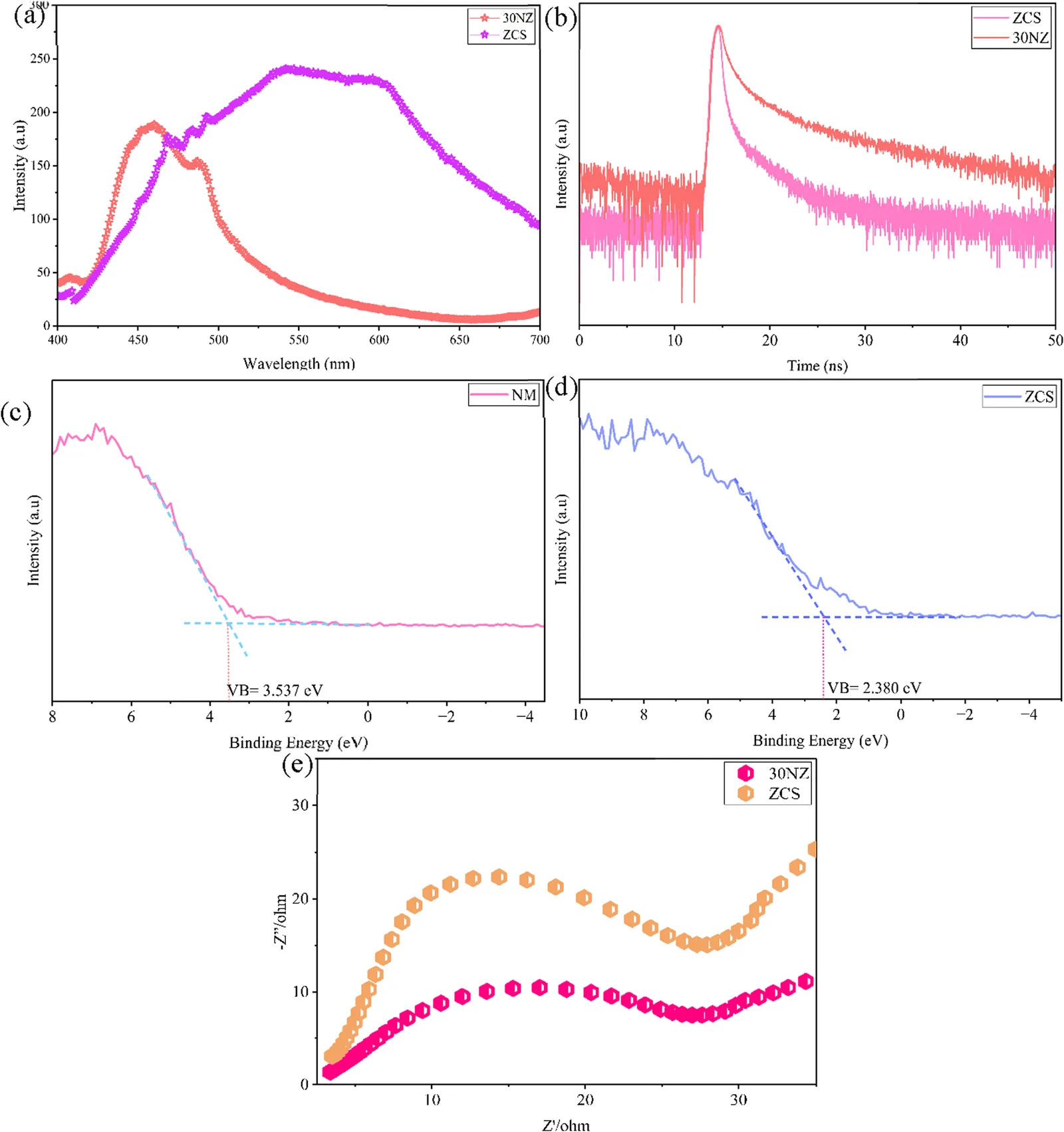
(a) PL analysis of ZCS and 30NZ. (b) Transient-state PL lifetime of ZCS and 30NZ. (c, d) XPS of valence band spectra of NM and ZCS. (e) EIS spectra of ZCS and 30NZ.

Figure 6(b) depicts the time-resolved photoluminescence analysis, which reveals the lifetime decay values. τ_1_ and τ_2_ correspond to fast decay component related to nonradiative recombination and slow decay component associated with radiative recombination of free electron and hole pairs. Radiative recombination, also known as band-to-band recombination, involves both VB and CB, where an electron falls back to VB from CB, releasing energy in the form of photons.^29^ Whereas nonradiative recombination dissipates energy as phonons during the recombination process, which hinders photon generation, hence diminishing the efficiency of the photocatalytic reduction process. As shown in Table S1, the τ_1_ value is significantly smaller than the τ_2_ value for both pristine (ZCS) and composite (30NZ), suggesting that radiative recombination dominated over the nonradiative recombination pathways. The prevalence of photon emission is consistent with the charge carrier utilization, hence contributing to the composite’s efficient photoreduction activity. The average lifetimes of ZCS and 30NZ were found to be 1.342 and 4.323 ns. The average lifetime of 30NZ is 3.22 times that of ZCS. The longer lifetime of 30NZ proves the enhanced charge separation and suppressed recombination of electrons and holes improved in the composite, consistent with efficient charge transfer.^30^

The valence band positions were determined by VB-XPS analysis, and the Mulliken electronegativity was used to calculate the conduction band positions using the energy band gap values from the Tauc plot, as shown in Figure 4(b). Figure 6(c,d) depicts the VB-XPS spectra of the pristine materials. The VB values obtained from the spectra were converted to the NHE scale using the formula *E*_VB‑NHE_ = φ + *E*_VB‑XPS_ – 4.44,^31^ and the calculated *E*_VB‑NHE_ were +3.297 V and +2.140 V for NM and ZCS, respectively. As shown in Table S2, the VB values calculated from VB-XPS analysis and Mulliken electronegativity are relatively similar; hence, the VB values were further used to estimate the band structure of the composite. Furthermore, the electrochemical impedance spectroscopy studies were carried out. As shown in Figure 6(e), the smaller Nyquist semicircle of 30NZ confirms the enhanced interfacial charge transfer and better carrier separation.^30^

### Photocatalytic Activity

The photocatalytic CO_2_ reduction under UV irradiation using NaOH as the solvent was performed as shown in Figure 7(a). NaOH was used as a solvent in this research work to improve CO_2_ dissolution in the liquid phase and perhaps increase CO_2_ molecule adsorption on the catalyst surface. Furthermore, research suggests that NaOH may improve electrical conductivity. Highly concentrated OH-ions in aqueous solution may function as potent hole scavengers and produce OH radicals, facilitating the flow of charges. According to the literature, the reaction between CO_2_ and NaOH may also produce intermediate products such as HCO^3–^ions, which are also beneficial for boosting the production of CH_3_OH when it gets photogenerated electrons on the surface of the photocatalyst. The flow rate of CO_2_ was kept at 35 mL min^–1^, and the distance between the light source and the glass reactor was maintained at 1.5–2 cm for better light irradiation on the surface of the solvent and the photocatalyst. The solution was stirred at 400–500 rpm for proper dissolution of CO_2_ in the solvent. The controlled experiment in the absence of CO_2_, light, and catalyst was performed just to confirm that the product formed is only due to the photocatalytic reduction process rather than external factors such as the solvent and catalyst. The pristine catalysts produced methanol at the rates of 4917.76 and 329.369 μmol g^–1^ h^–1^ for ZCS and NM, respectively, as shown in Table S3. It is found that the products formed are dependent on the NM content present in the catalyst. After the introduction of NM to ZCS, it is observed that as the content of NM is increased, ethanol is also formed alongside methanol as the products, and 30NZ gave the highest rate of ethanol of 3730.29 μmol g^–1^ h^–1^.

**Figure 7:**
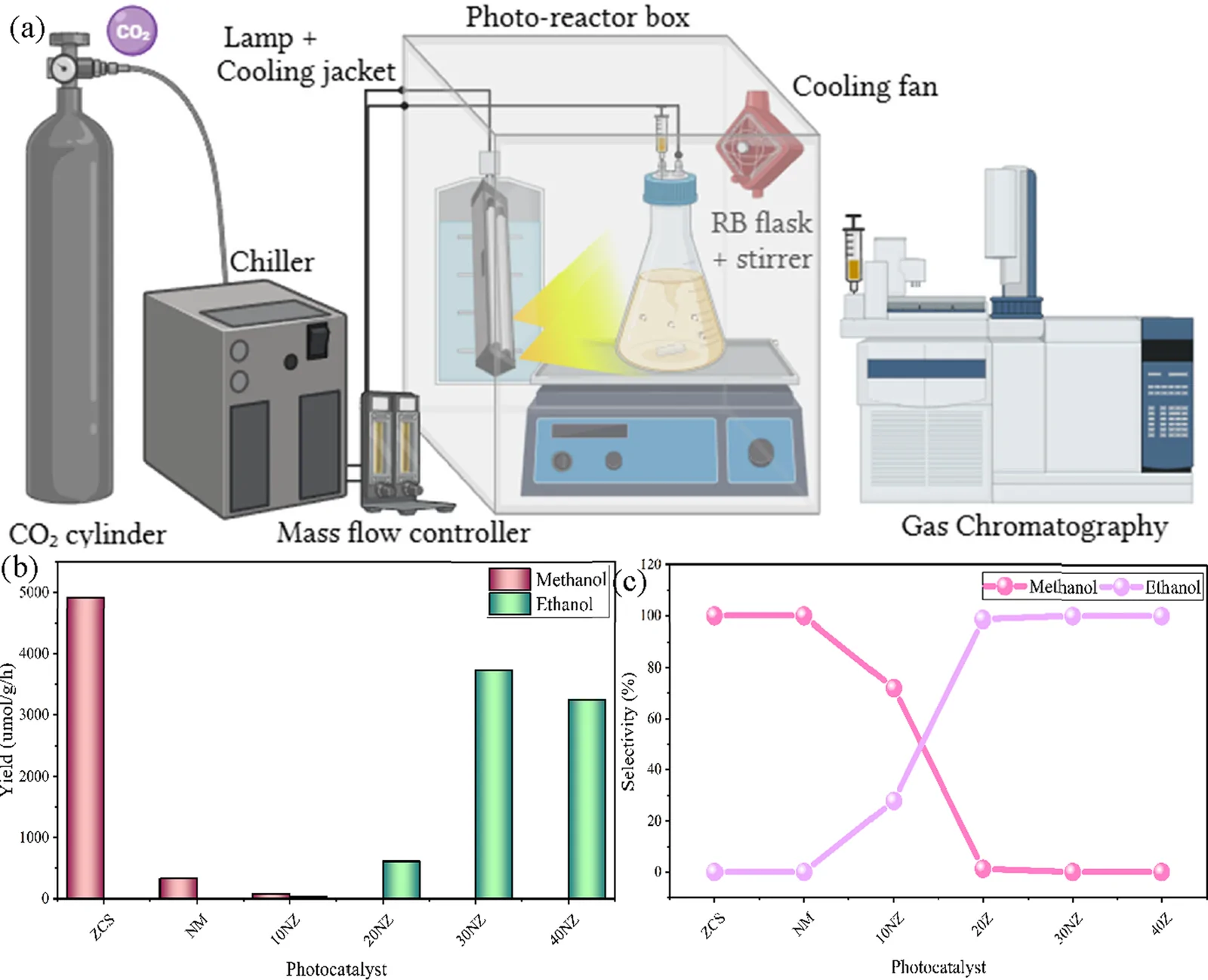
(a) Schematic diagram of photocatalytic setup with light illumination (image drawn by Thillai Lakshmi). (b) Graph representation of methanol and ethanol production using NZ composites. (c) Selectivity of NZ composites toward methanol and ethanol.

When 10% of NM is dispersed in the ZCS, i.e., 10NZ composite, methanol and ethanol are produced at the rates of 81.06 and 31.4905 μmol g^–1^ h^–1^, respectively, as shown in Figure 7(b). The methanol product formation for 20NZ decreases to 8.032 μmol g^–1^ h^–1^, whereas the ethanol production increases to 614.38 μmol g^–1^ h^–1^. Additionally, as Figure 7(c) illustrates, there is no methanol generation for 30% of NM, making it 100% selective for ethanol. The ethanol formation is found to decrease to 3246.46 μmol g^–1^ h^–1^ for 40% NM. Therefore, the product decreases as the NM content increases; this could be because the composite has a greater recombination rate or active sites are blocked. The yield of 30NZ is compared with the published photocatalysts in Table S4, where the NZ produced in the present study demonstrated higher photocatalytic activity than the other photocatalysts.

### Enhanced Photocatalytic Activity Mechanism

Figure 8 illustrates the NZ’s photocatalytic mechanism. The Mulliken electronegativity and the band gap values of the pristine compounds, which are calculated from a Tauc plot, are used to theoretically calculate the valence band (VB) and the conduction band (CB) in order to comprehend and analyze the mechanism and functioning of the photoreduction occurring on the composite. These calculations are given in the Supporting Information. The process is predicted based on the values of VB and CB displayed in Figure 8, and the potential electron and hole transfers associated with the synthesis of ethanol and methanol are depicted as a direct Z-scheme heterojunction.

**Figure 8:**
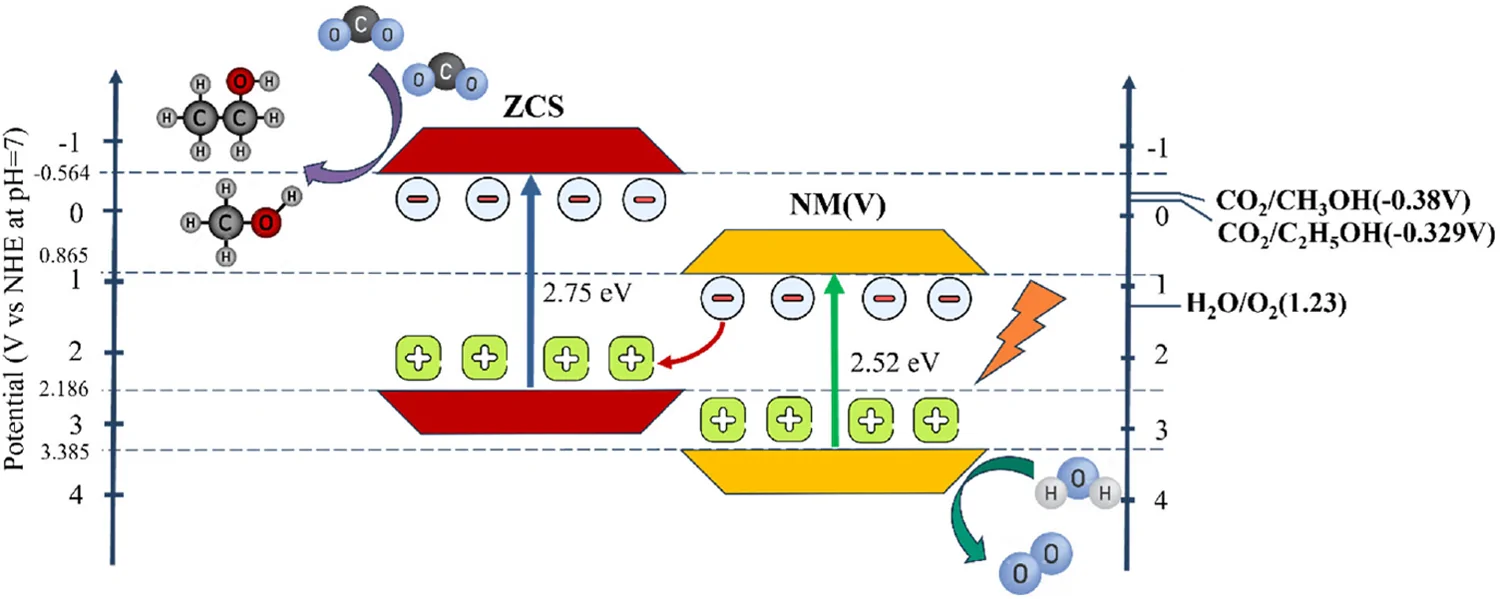
Schematic depiction of the photocatalytic CO_2_ reduction process and electron and hole transfer for the synthesis of methanol and ethanol in the NZ composite.

Electrons and holes are formed by light irradiation because the VB potential (3.385 eV vs NHE) of NM is more positive than the VB potential (2.186 eV vs NHE) of ZCS, and the CB potential (−0.564 eV vs NHE) of ZCS is more negative than the CB potential (0.865 eV vs NHE) of NM. The electron from the CB of NM recombines with the holes in the VB of ZCS, as shown in Figure 8. The residual strongly reducing electrons in CB (ZCS) and strongly oxidizing holes in VB (NM) are retained for redox reactions, where the electrons activate and reduce the CO_2_ molecules adsorbed and the holes participate in the oxidation of H_2_O to O_2_.^32^ It is evident from Figure 8 that the reduction potential of methanol and ethanol falls between the composites’ potential differences, theoretically demonstrating product formation.^33^ By prolonging the lifetime of photoinduced electrons and holes, as shown in Figure 6(b), this process prevents electron–hole recombination by suppressing nonradiative recombination, consistent with the Z-scheme configuration. Electrochemical impedance spectroscopy shows a significant decrease in charge transfer resistance for the composite under illumination, indicating that the NZ interface promotes fast carrier migration and effective separation of electrons and holes. Altogether, the band structure analysis, PL/TRPL analysis, and the electrochemical studies provide evidence that NM-ZCS follows the Z-scheme charge transfer mechanism.^31^ Hence, experimentally and theoretically, it is proven that methanol and ethanol production is possible utilizing the photoreduction process of reducing CO_2_. This work suggests several possible areas for future developments. Optimization of the light absorption qualities in the visible region can maximize the utilization of sunlight, changing the current system into a solar-powered CO_2_ conversion technology. Increasing the absorption of visible light and expanding the photoresponse toward longer wavelengths would improve solar-to-fuel efficiency and make it easier to operate under real-time, outdoor irradiation. The photocatalytic process can be methodically expanded to the selective generation of gaseous fuels, such as CH_4_, CO, and H_2_, in addition to liquid products like methanol and ethanol.

Photocatalytic CO_2_ reduction allows for the production of ethanol, which is a well-established chemical and fuel additive. This approach integrates carbon utilization with renewable energy. The NM/ZCS system could be designed into modular reactor units for decentralized CO_2_ valorization if the catalyst can be produced from abundant precursors, run at moderate temperatures, and connected to concentrated solar or artificial light sources. This work has to be studied for long-term stability, reactor engineering, and techno-economic analysis for it to become established for an ethanol production industry through CO_2_ reduction.

## Conclusion

In conclusion, highly active composites of Zn_0.2_Cd_0.8_S/NH_2_-MIL-125­(Ti) photocatalysts have been effectively produced using the simple in situ method. The composites demonstrated much greater photocatalytic activity for CO_2_ reduction-based methanol and ethanol generation when compared with pristine synthesized catalysts. It becomes apparent that the optimal loading percentage of NH_2_-MIL-125­(Ti) is approximately 30 wt %, and the corresponding rate of C_2_H_5_OH generation is 3730.29 μmol g^–1^ h^–1^. It is found that the significant improvement in photocatalytic activity can be attributed to the efficient charge separation and transfer between Zn_0.2_Cd_0.8_S and NH_2_-MIL-125­(Ti) simply by changing the loading of NH_2_-MIL-125­(Ti). The proposed Zn_0.2_Cd_0.8_S/NH_2_-MIL-125­(Ti) catalysts are a viable material for large-scale ethanol production sectors due to their strong photocatalytic activity and capacity to produce both ethanol and methanol.

## Supplementary Material



## Notes

Detailed synthesis procedure, PL lifetime fitting, estimation of VB and CB, photocatalytic activity results table, and comparative table of reported results (PDF)

## Abbreviations

C: Carbon
CO_2_: Carbon Dioxide
CO: Carbon Monoxide
HCOOH: Formic Acid
HCHO: Formaldehyde
CH_3_OH: Methanol
CH_4_: Methane
C_2_H_4_: Ethylene
C_2_H_6_: Ethane
CH_2_CH_3_OH: Ethanol
Cd: Cadmium
Cd­(CH_3_COO)_2_.2H_2_O: Cadmium Acetate Dihydrate
CdS: Cadmium Sulfide
CB: Conduction Band
DMF: Dimethylformamide
EDX: Energy-Dispersive X-ray Spectroscopy
EIS: Electrochemical Impedance Spectroscopy
FTIR: Fourier Transform Infrared Spectroscopy
FWHM: Full Width at Half Maximum
GHG: Greenhouse Gases
HUMO: Highest Occupied Molecular Orbital
JCPDS: Joint Committee on Powder Diffraction Standards
LUMO: Lowest Unoccupied Molecular Orbital
PL: Photoluminescence Analysis
N: Nitrogen
NaOH: Sodium Hydroxide
NM: NH_2_-MIL-125­(Ti)
NZ: NH_2_-MIL-125­(Ti)/Zn_0.2_Cd_0.8_S Composites
O: Oxygen
S: Sulfur
*S*_BET_: Surface Area
Ti: Titanium
TRPL: Time-Resolved Photoluminescence Spectroscopy
UV–vis: Ultraviolet–Visible
VB: Valence Band
XPS: X-ray Photoelectron Spectroscopy
XRD: X-ray Diffraction
Zn: Zinc
Zn­(CH_3_COO)_2_.2H_2_O: Zinc Acetate Dihydrate
Zn_0.2_Cd_0.8_S (ZCS): Zinc Sulfide
