# Supplementary material for: In Situ Fabrication of Metal Chalcogenide on Metal–Organic Frameworks for Enhanced Photocatalytic CO2 Reduction to Liquid Fuel
Source: ACS Omega. 2026 Aug 19;11(34):51202–14. doi: 10.1021/acsomega.6c03925 (PMC13625128; doi:10.1021/acsomega.6c03925)
Supplement: Supplementary file 1 [file ao6c03925_si_001.pdf]

**Supporting information**

# In-situ fabrication of metal chalcogenide on metal organic frameworks for enhanced photocatalytic $CO_2$ reduction to liquid fuel

*Thillai Lakshmi[1], Shanmuga Priya Selvanathan*[1], Lavanya Mulky[1], and Muhammad Tahir[2]*

[1]Manipal Institute of Technology, Manipal Academy of Higher Education, Manipal, Karnataka, India

[2]Chemical and Petroleum Engineering Department, UAE University, Al Ain, Abu Dhabi, P.O. Box 15551, United Arab Emirates

Corresponding author: shan.priya@manipal.edu

**Keywords:** MOF, chalcogenide, nanocomposite, conversion, ethanol

## Synthesis procedure of pristine ZCS and NM

### Synthesis of $Zn_{0.2}Cd_{0.8}S$ (ZCS)

The $Zn_{0.2}Cd_{0.8}S$ was synthesized with a few changes from this reported procedure(Tang et al., 2018). Accordingly, cadmium acetate, zinc acetate, and thiourea were dissolved in 60 ml of ethylene glycol containing polyvinylpyrrolidone and stirred at room temperature, and later sonicated for 5-10 minutes. After that, the solution is autoclaved at 150°C overnight. The final step was centrifuging the product to separate and collect it, and then repeatedly washing it with acetone and deionized water afterwards. The resultant product was vacuum dried at 60 °C. The resulting product is named ZCS.

### Synthesis of $NH_2$-MIL-125(Ti) (NM)

$NH_2$-MIL-125(Ti) was synthesized with slight modifications to the previously reported method(Zhou et al., 2024). Briefly, in a 1:1 ratio of methanol and DMF, 0.17M Titanium isopropoxide and 0.298M 2-Amino terephthalic acid were dissolved with rapid stirring. After stirring for 1-2 hours, it was sonicated for 5-10 minutes before being transferred and autoclaved at 150°C overnight. Subsequently, the mixture was cooled, washed with DMF and methanol, and vacuum dried at 60 °C. The resulting product is named NM.

**Table S1.** PL lifetime fitting results for ZCS and 30NZ

| **Sample** | **$A_1$** | **□$_1$** | **$A_2$** | **□$_2$** | **□$_{avg}$** | **□$_{avg\ (30NM)}$/ □$_{avg(ZCS)}$** |
|---|---|---|---|---|---|---|
| **ZCS** | 6151.7207 | 0.34536507 | 242.273 | 3.704232 | 1.3428308 | 1.0 |
| **30NZ** | 4324.9546 | 0.75636976 | 845.28638 | 6.4601116 | 4.3232994 | 3.219545 |

## Estimation of the VB and CB of ZCS and NM

The valence band (VB) and conduction band (CB) potential of a semiconductor can be theoretically calculated using Mulliken electronegativity and the bandgap of a semiconductor by the following formulas[3].

$E_{VB} = X - E_e + 0.5E_g$

$E_{CB} = E_{VB} - E_g$

where $E_{VB}$ and $E_{CB}$ are the top of the valence band and the bottom of the conduction band of the semiconductor, $E_g$ is the band gap energy, $E_e$ is the energy of free electrons on the hydrogen scale with a fixed value of 4.5 eV vs. NHE[3], [4], [5] and $X$ is the geometric mean of the Mulliken electronegativity of the constituent atoms in the semiconductor. The Mulliken electronegativity of an atom is the arithmetic mean of the first ionisation energy and the first electron affinity.

The detailed calculation steps for obtaining the Valence band and Conduction band of ZnCdS was illustrated as follows:

The first ionization energy of Zinc element (Zn): $I_{Zn}$ = 9.394197 eV,

The first electron affinity of Zinc element (Zn): $E_{Zn}$= -0.00 eV,

The Mulliken electronegativity of Zinc element (Zn): $\chi = \frac{1}{2}(I_{Zn} + E_{Zn}) = \frac{1}{2}(9.394197 + 0)$ = 4.697 eV.

The first ionization energy of Cadmium element (Cd): $I_{Cd}$ = 8.993820 eV,

The first electron affinity of Cadmium element (Cd): $E_{Cd}$= 0.00 eV,

The Mulliken electronegativity of Cadmium element (Cd): $\chi = \frac{1}{2}(I_{Cd} + E_{Cd}) = \frac{1}{2}(8.993820 + 0) = 4.497$ eV.

For Zn and Cd, the EA is often taken as ~0 eV in Mulliken calculations (they don't form stable atomic anions)

The first ionization energy of Sulphur element (S): $I_S$ = 10.3600167 eV,

The first electron affinity of Sulphur element (S): $E_S$ = 2.077 eV,

The Mulliken electronegativity of Sulphur element (S): $\chi = \frac{1}{2}(I_S + E_S) = \frac{1}{2}(10.3600167 + 2.077) = 6.2185$ eV.

Geometric mean= $\left(\prod_{i=1}^{n} \chi_i\right)^{\frac{1}{n}}$

The geometric mean of the Mulliken electronegativity for ZnCdS (Zinc cadmium sulphate) ($X$) is calculated as follows:

$X = ((X_{Zn})^{0.2} \times (X_{Cd})^{0.8} \times (X_S))^{1/2} = ((4.697)^{0.2} \times (4.497)^{0.8} \times (6.2185)^{1})^{1/2} = 5.311$ eV

The bandgap ($E_g$) of ZnCdS was obtained by the UV-visible diffuse reflectance measurement, and was determined to be 2.75 eV (Fig. 8(c)), i.e., $E_g$ (ZnCdS) = 2.75 eV.

Therefore, The Valence band of ZnCdS is calculated as follows:

$E_{VB} = X - E_e + 0.5E_g = 5.311 - 4.5 + 0.5 \times 2.75 = 2.186$ eV

The conduction band of ZnCdS is calculated as follows:

$E_{CB} = E_{VB} - E_g = 2.186 – 2.75 = -0.564$ eV

**The valence band and conduction band of ZnCdS were determined to be 2.186 eV and -0.564 eV, respectively.**

Then, for $NH_2$-MIL-125(Ti) ($C_{48}H_{34}N_6O_{36}Ti_8$), detailed calculation steps for obtaining the Valence band and Conduction band are illustrated as follows:

The first ionization energy of titanium element (Ti): $I_{Ti}$ = 6.8281 eV,

The first electron affinity of titanium element (Ti): $E_{Ti}$= 0.079 eV,

The Mulliken electronegativity of titanium element (Ti): $\chi = \frac{1}{2}(I_{Ti} + E_{Ti}) = \frac{1}{2}(6.8281 + 0.079) = 3.4536$ eV.

The first ionization energy of Nitrogen element (N): $I_N$ = 14.5341 eV,

The first electron affinity of Nitrogen element (N): $E_N$= 0.00 eV,

The Mulliken electronegativity of Nitrogen element (N): $\chi = \frac{1}{2}(I_N + E_N) = \frac{1}{2}(14.5341 + 0.00) = 7.2671$ eV.

The first ionization energy of hydrogen element (H): $I_H$ = 13.60 eV,

The first electron affinity of hydrogen element (H): $E_H$ = 0.756 eV,

The Mulliken electronegativity of hydrogen element (H): $\chi = \frac{1}{2}(I_H + E_H) = \frac{1}{2}(13.60+0.756) = 7.176$ eV.

The first ionization energy of carbon element (C): $I_C$ = 11.2603 eV,

The first electron affinity of carbon element (C): $E_C$ = 1.2621 eV,

The Mulliken electronegativity of carbon element (C): $\chi = \frac{1}{2}(I_C + E_C) = \frac{1}{2}(11.26+1.26) = 6.2612$ eV.

The first ionization energy of oxygen element (O): $I_O$ = 13.62 eV,

The first electron affinity of oxygen element (O): $E_O$ = 1.46 eV,

The Mulliken electronegativity of oxygen element (O): $\chi = \frac{1}{2}(I_O + E_O) = \frac{1}{2}(13.62+1.46) =$ 7.54 eV.

Geometric mean= $\left(\prod_{i=1}^{n} \chi_i\right)^{\frac{1}{n}}$

The geometric mean of the Mulliken electronegativity for $NH_2$-MIL-125(Ti) ($C_{48}H_{34}N_6O_{36}Ti_8$) ($X$) is calculated as follows:

$X = ((X_{Ti})^8 \times (X_N)^6 \times (X_C)^{48} \times (X_H)^{34} \times (X_O)^{36})^{1/132} = 6.625$ eV

The bandgap ($E_g$) of $NH_2$-MIL-125(Ti) was obtained by the UV-visible diffuse reflectance measurement, and was determined to be 2.52 eV (Fig. 8(c)), i.e., $E_g$ ($NH_2$-MIL-125(Ti)) = 2.52 eV.

Therefore, The Valence band of $NH_2$-MIL-125(Ti) is calculated as follows:
$E_{VB} = X - E_e + 0.5E_g = 6.625 - 4.5 + 0.5 \times 2.52 = 3.385$ eV

The conduction band of $NH_2$-MIL-125(Ti) is calculated as follows:

$E_{CB} = E_{VB} - E_g = 3.385 - 2.52 = 0.865$ eV

**The valence band and conduction band of $NH_2$-MIL-125(Ti) were determined to be 3.385 eV and 0.865 eV respectively.**

## Calculation of valence band energy (EVB) from XPS measurement:

The valence band energy ($E_{VB}$) of ZCS and NM was determined by valence band-XPS spectra (VB-XPS), as shown in Fig. 5 (c-d). The obtained potentials were converted to standard hydrogen potential ($E_{VB\text{-}NHE}$) using the following relationship:

$E_{VB\text{-}NHE} = \varphi + E_{VB\text{-}XPS} - 4.44$

where $\varphi$ is the work function of the XPS analyser (4.2 eV), and $E_{VB\text{-}XPS}$ is the value obtained from the VB-XPS spectrum. Thus, the calculated $E_{VB\text{-}NHE}$ was +3.297 V and +2.140 V for NM and ZCS.

**Table S2.** Comparison of VB values calculated using from Mulliken's electronegativity and VB-XPS analysis

| | Photocatalyst | Method | VB value (V vs NHE) | CB value (V vs NHE) | $E_g$ value |
|---|---|---|---|---|---|
| **Theoretical** | ZCS | Mulliken electronegativity | 2.186 | 0.564 | 2.75 |
| **Experimental** | | VB-XPS Analysis | 2.14 | - | - |
| **Theoretical** | NM | Mulliken electronegativity | 3.385 | 0.865 | 2.52 |
| **Experimental** | | VB-XPS Analysis | 3.297 | - | - |

**Table S3**. Summary of the photocatalytic activity of the synthesized catalysts

| Photocatalyst | Solvent used | Solvent volume (mL) | Flow rate of $CO_2$ (mL/min) | Light source(W) | Products | Yield(μmol $g^{-1}h^{-1}$) |
|---|---|---|---|---|---|---|
| ZCS | 0.1 M NaOH | 100 | 35 | UV | Methanol | 4917.76 |
| NM | 0.1 M NaOH | 100 | 35 | UV | Methanol | 329.369 |
| 10NZ | 0.1 M NaOH | 100 | 35 | UV | Methanol<br>Ethanol | 81.0699<br>31.4905 |
| 20NZ | 0.1 M NaOH | 100 | 35 | UV | Methanol<br>Ethanol | 8.0329<br>614.388 |
| 30NZ | 0.1 M NaOH | 100 | 35 | UV | Ethanol | 3730.29 |
| 40NZ | 0.1 M NaOH | 100 | 35 | UV | Ethanol | 3246.49 |

**Table S4.** Comparative table of photocatalytic activity of $NH_2$-MIL-125(Ti)/$Zn_{0.2}Cd_{0.8}S$ with reported photocatalysts

| **Photocatalyst** (g) | **Solvent used (mL)** | **Light source(W)** | **Products** | **Yield(μmolg$^{-1}$h$^{-1}$)** | **References** |
|---|---|---|---|---|---|
| $Zn_{0.2}Cd_{0.8}S$ (0.1) | 0.1 M NaOH (100) | 250W UV lamp | Methanol | 4917.76 | This work |
| $NH_2$-MIL-125(Ti)/$Zn_{0.2}Cd_{0.8}S$ (0.1) | 0.1 M NaOH (100) | 250W UV lamp | Ethanol | 3730.29 | This work |
| CuCo-ZIF@g-$C_3N_4$ (-) | NaOH (-) | Visible Light | Methanol | 1018.65 | [6] |
| $Zn_{0.2}Cd_{0.8}S$/ g-$C_3N_4$ (0.01) | $H_2O$ (100) | 300 W Xe lamp | Methanol | 700.5 | [7] |
| $Cu_2O$/Pt/$NH_2$-MIL-125(Ti) (0.1) | DI $H_2O$ (100) | 300 W Xe lamp | Methanol<br>Ethanol | 192<br>307 | [8] |
| ZnS/CQDs/$NH_2$MIL-125(Ti) (0.1) | DI $H_2O$ (100) | 300 W Xe lamp | Methanol<br>Ethanol | 169.19<br>188.23 | [2] |
| $Cd_{0.2}Zn_{0.8}S$/ UiO-66-$NH_2$ (0.05) | 0.1 M $Na_2S$ + 0.1 M $Na_2SO_3$ (100) | 300 W Xe lamp | Methanol<br>Hydrogen | 6.8<br>5846.5 | [9] |

**References**


[1] L. Tang, L. Kuai, Y. Li, H. Li, Y. Zhou, and Z. Zou, "Zn x Cd1-xS tunable band structure-directing photocatalytic activity and selectivity of visible-light reduction of CO2 into liquid solar fuels," *Nanotechnology*, vol. 29, no. 6, Jan. 2018, doi: 10.1088/1361-6528/aaa272.

[2] J. Zhou, Q. Jia, L. Gong, S. Zhang, X. Zhou, and H. Song, "Enhanced Z-scheme ZnS/NH2−MIL-125(Ti) photocatalysts with biomass-derived carbon quantum dots for

CO2 reduction,” *Molecular Catalysis*, vol. 552, Jan. 2024, doi: 10.1016/j.mcat.2023.113715.

[3] H. Shi, G. Chen, C. Zhang, and Z. Zou, “Polymeric g-C3N4 coupled with NaNbO3 nanowires toward enhanced photocatalytic reduction of CO2 into renewable fuel,” *ACS Catal*, vol. 4, no. 10, pp. 3637–3643, Oct. 2014, doi: 10.1021/cs500848f.

[4] Y. Cheng, L. He, G. Xia, C. Ren, and Z. Wang, “Nanostructured g-C3N4/AgI composites assembled by AgI nanoparticles-decorated g-C3N4 nanosheets for effective and mild photooxidation reaction,” *New Journal of Chemistry*, vol. 43, no. 37, pp. 14841–14852, 2019, doi: 10.1039/C9NJ02725D.

[5] B. Lin, G. Yang, B. Yang, and Y. Zhao, “Construction of novel three dimensionally ordered macroporous carbon nitride for highly efficient photocatalytic activity,” *Appl Catal B*, vol. 198, pp. 276–285, 2016, doi: https://doi.org/10.1016/j.apcatb.2016.05.069.

[6] K. Boukayouht, N. U. M. Nor, Y. Ait-Khouia, W. L. Queen, N. A. Saidina Amin, and S. El Hankari, “Enhanced Photocatalytic $CO_2$ Reduction to Methanol over Eco-Friendly CuCo-ZIF@g-$C_3N_4$ Synthesized from Recyclable Resources,” *Inorg. Chem.*, vol. 64, no. 9, pp. 4308–4319, Mar. 2025, doi: 10.1021/acs.inorgchem.4c04838.

[7] Y. Zhang *et al.*, “Construction of Zn0.2Cd0.8S/g-C3N4 nanosheet array heterojunctions toward enhanced photocatalytic reduction of CO2 in visible light,” *Colloids Surf. A Physicochem. Eng. Asp.*, vol. 655, Dec. 2022, doi: 10.1016/j.colsurfa.2022.130240.

[8] Q. Jia, J. Zhou, L. Gong, L. Wang, X. Ma, and Y. Zhao, “Z-scheme heterostructure of Cu2O/Pt/NH2-MIL-125(Ti) for photocatalytic CO2 reduction,” *Molecular Catalysis*, vol. 530, Sep. 2022, doi: 10.1016/j.mcat.2022.112599.

[9] Y. Su, Z. Zhang, H. Liu, and Y. Wang, “Cd0.2Zn0.8S@UiO-66-NH2 nanocomposites as efficient and stable visible-light-driven photocatalyst for H2 evolution and CO2 reduction,” *Appl. Catal. B*, vol. 200, pp. 448–457, Jan. 2017, doi: 10.1016/j.apcatb.2016.07.032.
